# Genotyping-by-sequencing of a melon (*Cucumis melo* L.) germplasm collection from a secondary center of diversity highlights patterns of genetic variation and genomic features of different gene pools

**DOI:** 10.1186/s12864-016-3429-0

**Published:** 2017-01-09

**Authors:** Stefano Pavan, Angelo Raffaele Marcotrigiano, Elena Ciani, Rosa Mazzeo, Vito Zonno, Valentino Ruggieri, Concetta Lotti, Luigi Ricciardi

**Affiliations:** 1 Department of Soil, Plant and Food Sciences, University of Bari “Aldo Moro”, Via Amendola 165/A, 70126 Bari, Italy; 2 Department of Biosciences, Biotechnologies and Biopharmaceutics, University of Bari “Aldo Moro”, Via Amendola 165/A, 70126 Bari, Italy; 3 Sequentia Biotech, Barcelona, Spain; 4 Department of the Sciences of Agriculture, Food and Environment, University of Foggia, via Napoli 25, I-71100 Foggia, Italy

**Keywords:** *Cucumis melo*, Genetic diversity, Genomics, Genotyping-by-sequencing, Molecular breeding

## Abstract

**Background:**

Melon (*Cucumis melo* L.) is one of the most important horticultural species, which includes several taxonomic groups. With the advent of next-generation sequencing, single nucleotide polymorphism (SNP) markers are widely used in the study of genetic diversity and genomics.

**Results:**

We report the first successful application of genotyping-by-sequencing (GBS) technology in melon. We detected 25,422 SNPs by the analysis of 72 accessions collected in Apulia, a secondary centre of diversity in Southern Italy. Analyses of genetic structure, principal components, and hierarchical clustering support the identification of three distinct subpopulations. One of them includes accessions known with the folk name of ‘*carosello*’, referable to the *chate* taxonomic group. This is one of the oldest domesticated forms of *C. melo*, once widespread in Europe and now exposed to the risk of genetic erosion. The second subpopulation contains landraces of ‘*barattiere*’, a regional vegetable production that was never characterized at the DNA level and we show was erroneously considered another form of *chate* melon. The third subpopulation includes genotypes of winter melon (*C. melo* var. *inodorus*). Genetic analysis within each subpopulation revealed patterns of diversity associated with fruit phenotype and geographical origin. We used SNP data to describe, for each subpopulation, the average linkage disequilibrium (LD) decay, and to highlight genomic regions possibly resulting from directional selection and associated with phenotypic variation.

**Conclusions:**

We used GBS to characterize patterns of genetic diversity and genomic features within *C. melo*. We provide useful information to preserve endangered gene pools and to guide the use of germplasm in breeding. Finally, our findings lay a foundation for molecular breeding approaches and the identification of genes underlying phenotypic traits.

**Electronic supplementary material:**

The online version of this article (doi:10.1186/s12864-016-3429-0) contains supplementary material, which is available to authorized users.

## Background

Melon (*Cucumis melo* L., 2n = 2 × = 24) is one of the most important vegetables worldwide. Current world production of melon is over 31 million tons [1] and is prevalently located in Mediterranean and East Asian countries. The intraspecific classification of *C. melo* has been revised several times. The most recent one [2] includes two subspecies, *melo* and *agrestis*, and 15 groups or *varietas*: *acidulous*, *chinensis*, *conomon*, *makuwa* and *momordica* (ssp. *agrestis*), and *adana*, *ameri*, *cantalupensis*, *chandalak*, *chate*, *dudaim*, *flexuosus*, *inodorus*, *reticulatus* and *tibish* (ssp. *melo*). The groups *cantalupensis* and *inodorus* have the greatest commercial interest [3].

The Apulia region in Southern Italy is an important secondary centre of diversity for *C. melo*. Besides several landraces of winter melon (*C. melo* var. *inodorus*), the *chate* melon is still grown there as a last relic of a former wider cultivation in Europe, and is known with the folk name of ‘*carosello’* [4, 5]. Its fruits, cylindric in shape and typically covered by long trichomes, are harvested unripen and used raw in replacement of cucumbers. Laghetti et al. [6] assigned another typical Apulian vegetable production, referred to as ‘*barattiere’*, to the *chate* group. However, it is readily distinguishable from ‘*carosello*’ by its rounder shape and shorter trichomes. Together, germplasm of ‘*carosello’* and ‘*barattiere’* is estimated to be cultivated over a modest area (about 100 ha), and thus is seriously exposed to the risk of genetic erosion [7].

The study of genetic diversity is of utmost importance to address conservation programmes contrasting the erosion of cultivated gene pools and to guide the use of germplasm in breeding. With the advent of next-generation sequencing (NGS), single nucleotide polymorphism (SNP) markers are commonly used to describe genetic diversity, as they are present in a large number and merge excellent attributes such as wide genomic distribution, high reproducibility and co-dominant inheritance. Today, the genotyping-by-sequencing (GBS) assay is considered one of the most convenient approach for simultaneous large-scale SNP discovery and genotyping [8] and is widely employed to detect polymorphisms in plant species with sequence information [9, 10]. There are no reports of the application of GBS in melon, although its genome is publicly available [11].

Here, we used a GBS approach for the genetic characterization of Apulian germplasm of *C. melo*. Besides local landraces of winter melon, several accessions of ‘*carosello*’ and ‘*barattiere*’ were included in the analysis, and were shown to form clearly distinct gene pools. In addition, SNP data were used to provide information on linkage disequilibrium decay, to highlight regions putatively subjected to directional selection and to carry out a genome-wide association study (GWAS).

## Results

### Library sequencing and SNP calling

Sequencing of a 72-plex GBS library yielded about 160 million good barcoded reads, corresponding to an average of 2,2 million reads/sample. About 66% of the reads were successfully mapped onto the melon genome. The TASSEL-GBS pipeline [9] yielded 25,422 polymorphisms, supported by an average depth of 67. About 50% of the SNPs fell in intergenic regions, 24% in introns, 20% in exons and 6% in UTR regions. SNPs in the coding regions lead to 44.2% of synonymous, 52.2% of missense and 3.6% of non-sense mutations. The observed transition/transvertion ratio was 1.71. All the accessions contained less than 20% of missing data across the whole dataset of SNP loci.

### Genetic structure

**Fig. 1:**
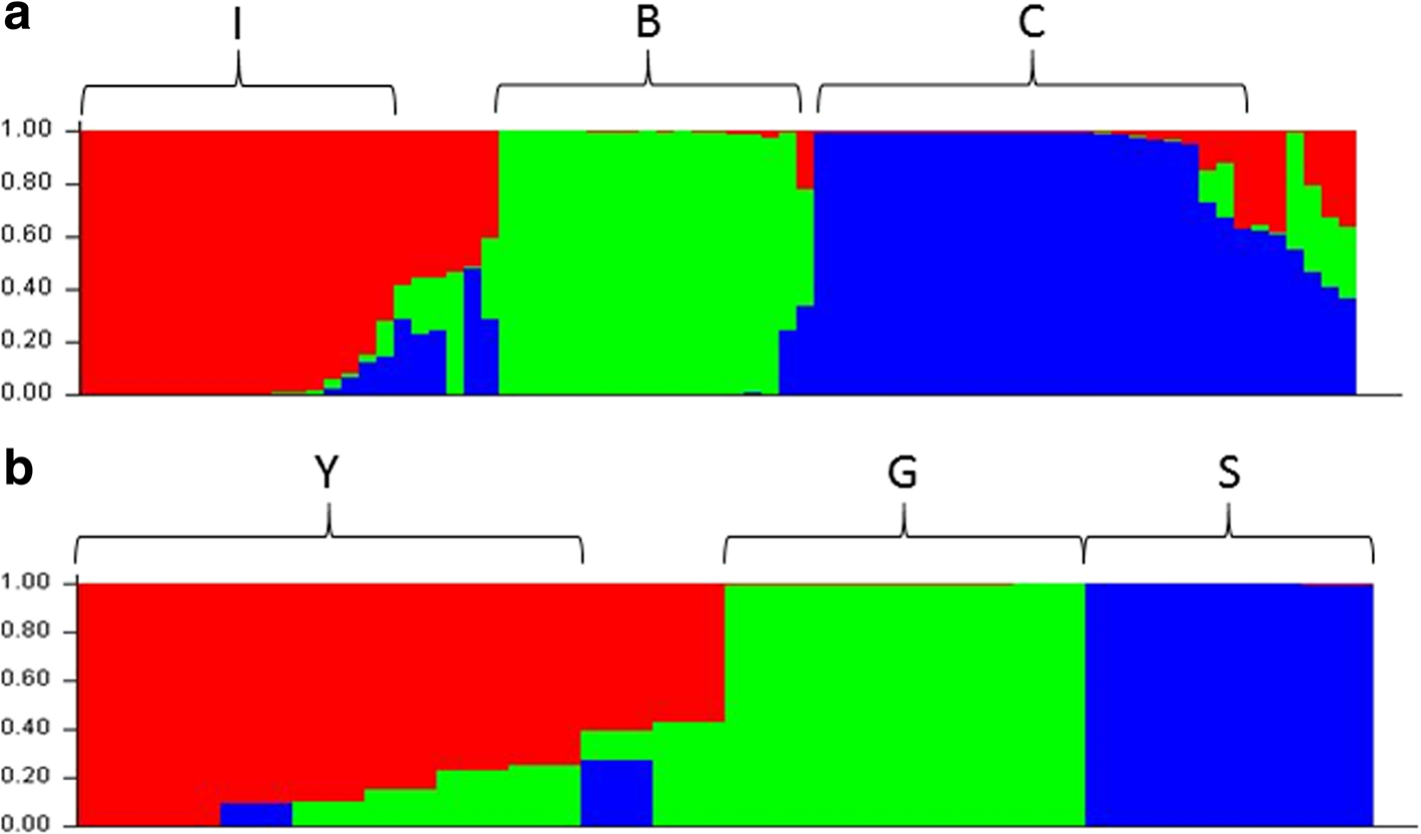
Population structure analysis. Results are shown for the minimum number of subpopulations (K) which sufficiently define genetic variation, as inferred by the estimation of the ΔK parameter. Each individual is represented by a vertical line, which is partitioned into coloured segments whose length depends on the estimated membership fraction (q) in each subpopulation. Individuals are assigned to a specific subpopulation when the highest q is higher than 0.6 (**a**) Genetic structure of the *C. melo* germplasm collection used in this study. The subpopulations *I*, *C*, and *B*, which refer to the types *inodorus*, ‘*carosello’* and ‘*barattiere’*, respectively, are indicated. **b** Genetic structure of the subpopulation *I*. The three subgroups are named *Y*, *G* and *S* as they contain accessions with *yellow*, *green* and speckled fruit rind, respectively

**Fig. 2:**
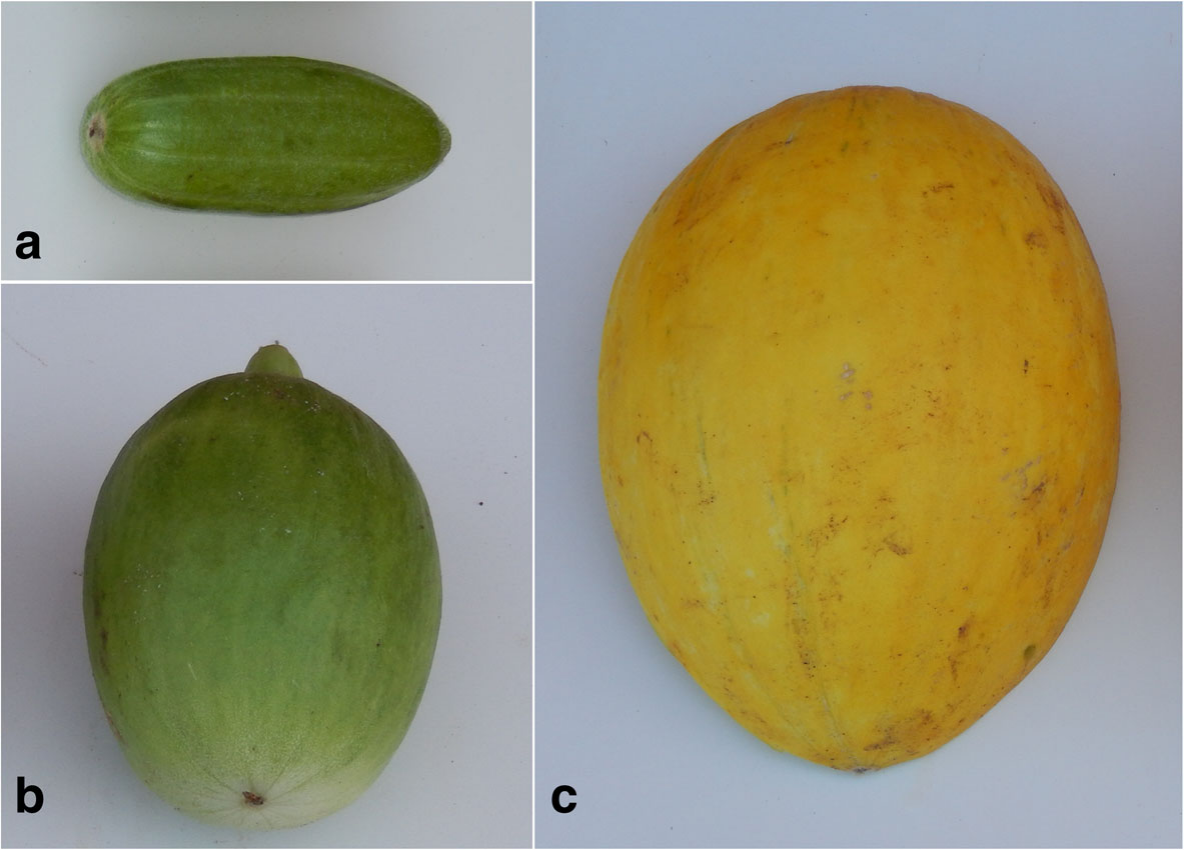
Typical fruit phenotype of **a** ‘*carosello*’, ‘*barattiere*’ (**b**) and ‘*inodorus*’ (**c**) accessions cultivated in the Apulian center of diversity

An admixture-based clustering model implemented in the software STRUCTURE [12] was used to infer the genetic structure of a germplasm collection representative of the genetic variation of *C. melo* cultivated in Apulia (Additional file 1). Prior to analysis, biallelic SNPs were filtered with stringent parameters (MAF > 0.05, call rate > 80%, and proportion of heterozygous < 50%), resulting in 8,012 high-quality polymorphisms scattered throughout the 13 *C. melo* pseudochromosomes present in the melon 3.5.1. genome assembly [13] (Additional file 2). Moreover, as the model assumes independence of loci, the SNP dataset was pruned on the basis of estimates of pairwise linkage disequilibrium between adjacent markers. A model with three subpopulations (K = 3) (Fig. 1a) was the most suitable for the data, as inferred by the estimation of the ΔK parameter [14] (Additional file 3). Remarkably, the distribution of the accessions in the three subpopulations fully matched with the phenotypic classification in *inodorus*, ‘*carosello*’ and ‘*barattiere*’ (Fig. 2). Thus, the subpopulations were named I, C and B, respectively (Fig. 1a). About 15% of the accessions was classified of admixed ancestry, as the highest estimate of membership coefficient was lower than 0.6. Overall estimates of the pairwise fixation index (F_ST_ values) were 0.47 between B and I (95% confidence boundaries: 0.46–0.48), 0.35 between B and C (95% confidence boundaries: 0.34–0.36) and 0.27 between C and I (95% confidence boundaries: 0.26–0.28).

Genetic structure was also studied within each subpopulation. To this aim, the SNP dataset was filtered separately for groups of accessions, resulting in the detection of 10,725 polymorphisms in C, 7,013 in I and 5,353 in B. A model with K = 3 best explained stratification within I (Fig. 1b). Each group included accessions with different fruit rind: yellow, green and speckled (Additional file 4). A model with K = 2 was the most likely for C, however no correlation was found between each of the two groups and specific features of the accessions (data not shown). Finally, no stratification was detected by the analysis of the subpopulation B.

### Genetic relationships among individual accessions

**Fig. 3:**
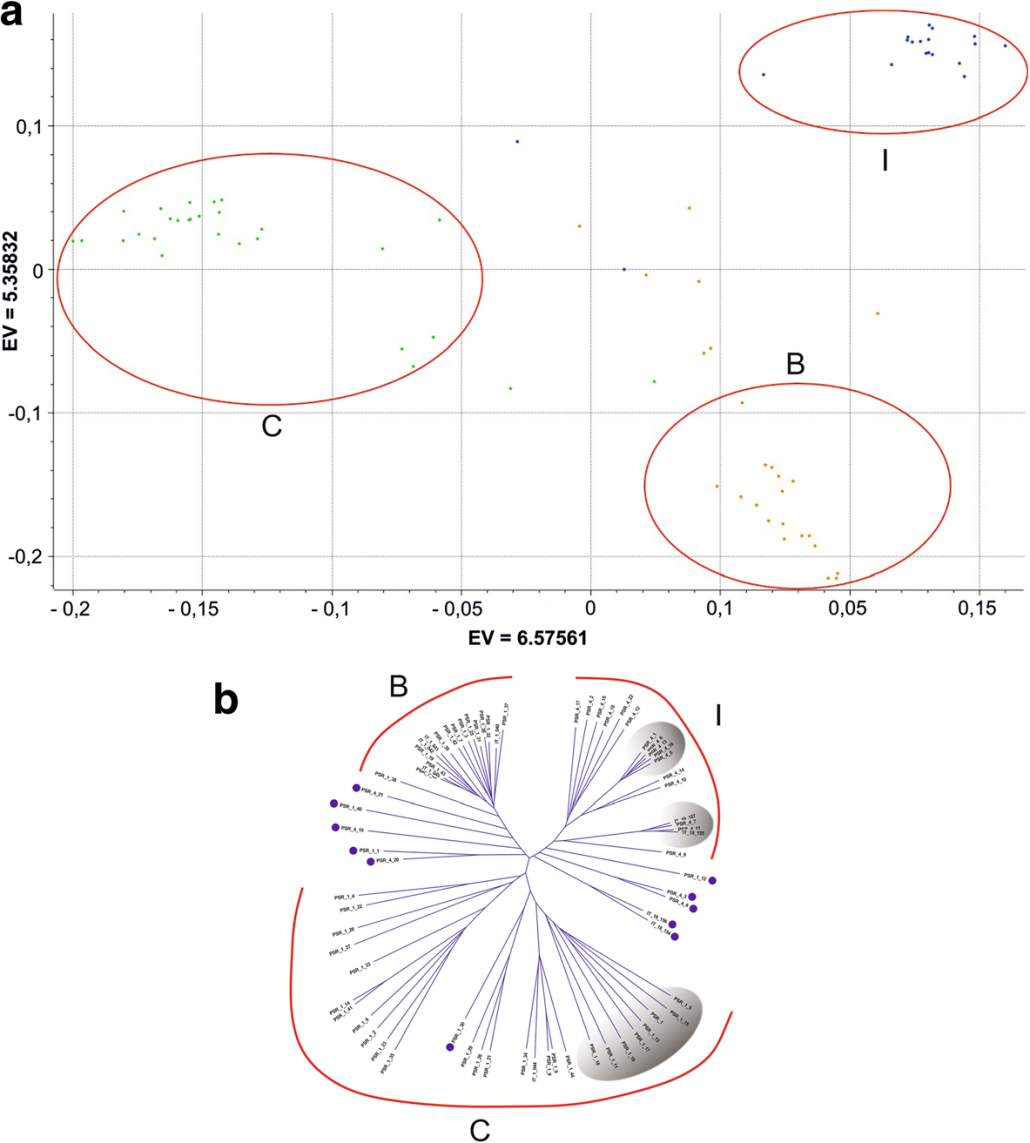
Genetic relationships within the germplasm collection used in this study. **a** Principal component analysis (PCA) plot. Different colors represent accessions phenotypically classified as *inodorus* (*orange*), ‘*carosello’* (*green*) and ‘*barattiere’* (*blue*). Circles delimit groups corresponding to the subpopulations *C*, *B* and *I* identified by structure analysis (**b**) Neighbour-Joining cladogram. Accessions assigned to the *C*, *B* and *I* subpopulations are highlighted (*red lines*). Admixed accessions are indicated with dots. Shaded areas indicate the *I* genetic clusters including accessions with *green* and speckled fruit rind and the *C* genetic cluster encompassing all the accessions collected in Southern Apulia

Principal component analysis (PCA) and Neighbour-Joining clustering were performed to identify patterns of genetic variation among individual accessions. Genotypes of I, C and B formed three PCA distinct groups, while admixed accessions were scattered at the center of the PCA plot (Fig. 3a). Further PCAs were performed to understand genetic relationships within each subpopulation. PCA within I revealed the occurrence of three groups, corresponding to accessions with yellow, green and speckled fruit rind (Additional file 5). Interestingly, the upper-right panel of the C PCA plot contained all the accessions collected in the province of Lecce, in the southern part of Apulia (Additional file 6). No clear correlation was found between PCA patterns and specific features of B accessions (data not shown).

Neighbour-Joining clustering substantiated the results obtained with structure analysis and PCA. Three main nodes separated the subpopulations I, C and B. Within I, two clusters enclosed accessions with green and speckled rind, respectively; within C, a cluster contained all the accessions collected in Southern Apulia (Fig. 3b).

### LD decay

**Fig. 4:**
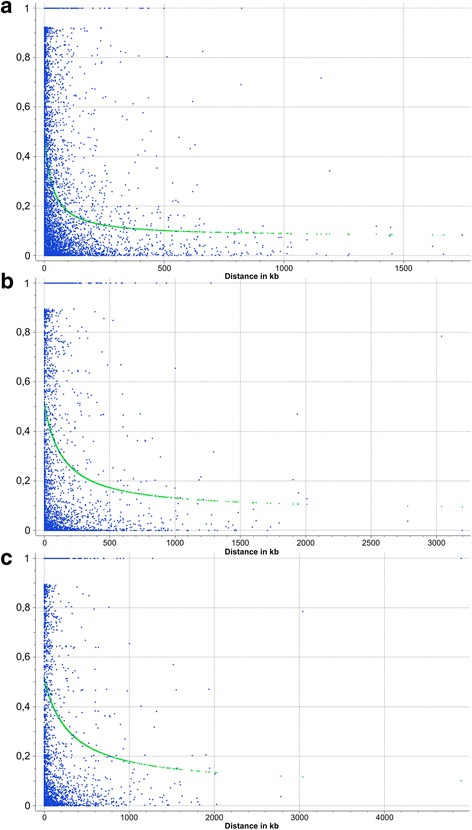
Average LD decay (r^2^) estimated in the *C. melo* subpopulations *C* (**a**), *B* (**b**) and *I* (**c**)

Having shown that the *C. melo* germplasm collection used in this study is stratified in three distinct subpopulations, we decided to estimate LD decays for each of them separately (Fig. 4). The fastest LD decay was displayed by the subpopulation C, as r^2^ reached the threshold of 0.2 after 72 Kb. Conversely, the slowest decay was associated with the subpopulation I, in which the same threshold r^2^ value corresponded to a distance of 774 kb. The subpopulation B showed an intermediate behavior (*r* ^2^ = 0.2 after 285 kb).

### Genomic scan for selection signatures

**Fig. 5:**
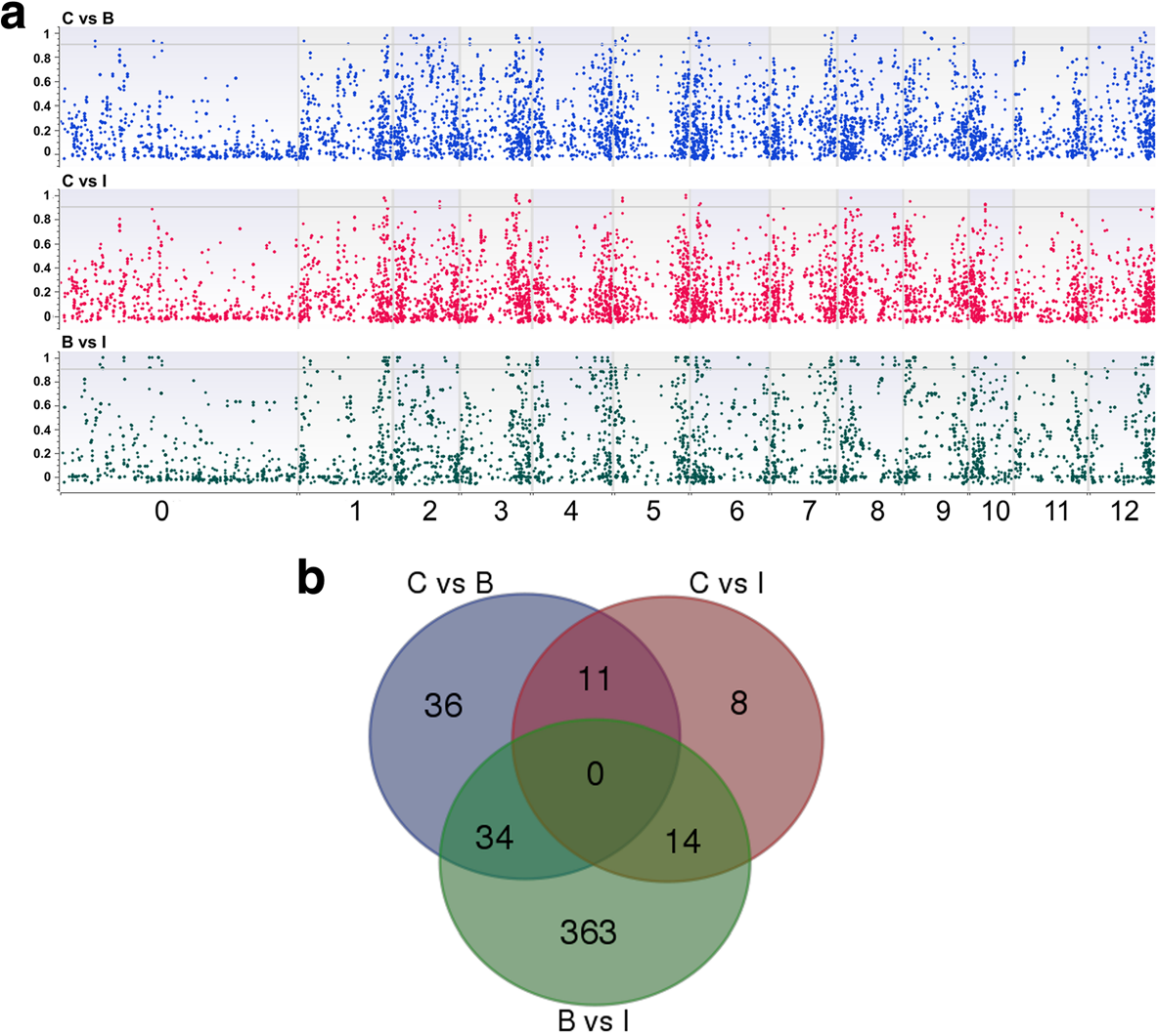
Analysis of single-loci pairwise F_ST_ estimates among the subpopulations *C*, *B* and *I*. **a** Genomic distribution of 8,012 SNP loci identified in this study in function of pairwise F_ST_ estimates. The *horizontal line* on each graph defines highly polymorphic loci associated with F_ST_ > 0.9 (**b**) Venn diagram of highly polymorphic loci (F_ST_ > 0.9)

We estimated the pairwise fixation index (F_ST_) parameter at individual SNPs, in order to identify loci putatively subjected to different selection pressures in the subpopulations I, C and B. F_ST_ approaches the upper limit of 1 when two subpopulations tend to fix opposite alleles. Thirty-three highly divergent loci (F_ST_ > 0.9) were identified between the subpopulations C and I, 81 between C and B and 411 between B and I (Fig. 5 and Additional file 7). Two adjacent divergent loci on chromosome 6 (6:1819634-SNV and 6:1819635-SNV), positioned within the putative *HOPM interactor 7* homolog *MELO3C006224T1*, define alleles which are private to the B group (F_ST [C vs B]_ = 1 and F_ST [B vs I]_ = 1). Similarly, the intergenic locus 5:27200654-SNV on chromosome 5 defines an allele private to the I group (F_ST [B vs I]_ = 1 and F_ST [C vs I]_ = 1) (Additional file 7). Several clusters of at least two consecutive markers displaying F_ST_ > 0.9 were observed for all the pairwise comparisons: 70 between B and I, 16 between C and B and 9 between C and I (Additional file 8). The largest cluster refers to the B vs I comparison and includes 31 loci spanning an interval of 41,791 bp on chromosome 1 (Additional file 8).

### Genome-wide association study

A GWAS assay was performed to search for loci associated with the following morpho-agronomic traits: flowering time both for male and perfect flowers (FLTM and FLTP), seed length (SL), seed width (SW) and 100 seed weight (SWe). FLTM was significantly associated with two SNP loci, 2:689400-SNV and 8:24132601-SNV, on chromosome 2 and 8, respectively (Additional file 9). The former is located within the gene *MELO3C015310*, encoding a putative rubber elongation factor/small rubber particle protein (Ref/SRPP), while the latter is located in an intergenic region. Two significant associations were also found for SW (Additional file 10), referable to the intergenic locus 2:19079933-SNV, positioned on chromosome 2, and the chromosome 6 locus 6:2464455-SNV, located within the gene MELO3C006314 encoding a putative 60S ribosomal protein L13-a2.

## Discussion

Here we report the first application of GBS, a low-cost and high-throughput approach widely used to explore genetic diversity in cultivated species [15], for the characterization of *C. melo* germplasm. More than 25K polymorphisms were detected, suggesting that GBS could be conveniently used in this species for further characterization of collections and other genomic studies.

Structure analysis indicates that three clearly distinct subpopulations define the genetic variation of the *C. melo* germplasm cultivated in Apulia, a secondary center of diversity in Southern Italy. These include accessions of winter melon and entries of morphotypes known with the folk names of ‘*carosello*’ and ‘*barattiere*’. The admixed accessions detected in this study, representing 15% of the collection, might originate from cross-pollination events during on-farm seed production, ultimately leading to genomic introgressions.

The *chate* melon has a centuries-old history of cultivation, starting at least from the ancient Egypt [16]. In Italy, it was presumably present already during the Roman Empire, although the first written documents indicating its cultivation date back to the Middle Age [17, 18]. In contrast with its past wide distribution, today *chate* melons are found in a few local markets. This study, providing a first molecular characterization of the *chate* germplasm, might be of great interest to preserve this taxonomic group from genetic erosion. Interestingly, PCA and hierarchical clustering analyses indicate that patterns of genetic diversity of Apulian *chate* melons also depend on their geographical origin, and this information could be conveniently used to orient germplasm conservation actions and breeding.

Prior to this study, landraces known as ‘*barattiere*’, despite being morphologically distinct from those of ‘*carosello*’, were also assigned to the *chate* taxonomic *varietas* [6]. The results of this study question this notion, as the B and C subpopulation form clearly distinct genetic pools. Moreover, the genetic distance between B and C (estimated by F_ST_) is higher than the distance observed between melons belonging to the different *varietas* *chate* (‘*carosello*’) and *inodorus* (winter melon). Possibly, the confusion between the two types arose from their common use as unripen fruits. It would be extremely interesting to compare, at the genomic level, ‘*barattiere*’ with all the intraspecific *varietas* reported for *C. melo* [2]. This might provide insights into the taxonomy of *C. melo* and its cultivation history.

The *inodorus* group is traditionally cultivated in Southern Italy, where it is appreciated for its long storability that allows consumption during the whole autumn and winter [19]. Here, we show that the distribution of molecular diversity of *inodorus* accessions is associated with the rind phenotype. This finding is consistent with a previous cluster analysis carried out on Spanish and Eurasian germplasm [3]. Yellow and green rind accessions collected in this study are morphologically similar to the Spanish ‘Amarillo’ and ‘Tendral’ types, respectively. Close genetic relationships between Italian and Spanish *inodorus* germplasm, which might be unveiled by future studies, are likely to occur as Southern Italy experienced a long Spanish domination during the Early Modern period.

Another aim of this work was to study the average LD decay in *C. melo* germplasm, an information of great importance for GWAS. Tomason et al. [20] previously used genome-wide data for the quantification of average LD decay in *C. melo*. However, these authors pooled several taxonomic groups and used simple sequence repeat (SSR) markers, therefore their results are not comparable to those reported in this study. The subpopulation I displayed a much slower LD decay than C and B. This might be explained by the reasonable assumption that winter melon experienced a stronger anthropic selection pressure than ‘*carosello*’ and ‘*barattiere*’, which led to the fixation of a higher number of LD blocks. The slow LD decay in the *inodorus* group is ideal to identify markers associated with favourable traits, and thus assisted selection, as it allows for an efficient coverage of the genome with a relatively low number of markers. On the other hand, long LD blocks may represent a limiting factor when association studies are aimed to fine-map genes of interest.

An additional goal of this study was to detect putative selection signatures in the genome of the three *C. melo* subpopulations characterized by structure analysis. Thus, several highly divergent SNP loci were identified by the estimation of pairwise F_ST_ values. As we showed that LD decays at a low rate in *C. melo*, the chance is high that these loci are not the real targets of selection, but are rather in association with them. Unfortunately, knowledge on the molecular basis underlying phenotypic variation in *C. melo* is still scarce. Moreover, the B and C groups, which are of local commercial interest, lack a thoroughly phenotypic characterization. Filling these gaps might thus help to associate high F_ST_ loci identified in this study with specific events of selection.

Previous works successfully used populations of moderate size for GWAS [21–23]. Therefore, we decided to use our germplasm collection for a medium-resolution association study, whose results might be integrated by further investigations taking into account larger samples and replicated trials. To reduce the amount of false-positives, we used a weighted mixed linear model (MLM) taking into account kinship and genetic structure (K + Q), which was proven useful in GWAS [24–27]. Moreover, to increase statistical stringency, we considered loci with high minimum allele frequency (>10%) and used the Bonferroni correction. Significant associations were detected for seed width and flowering time. These two traits are well-studied in plants, although little information is so far available in melon [28, 29]. Interestingly, a putative *GID1c* gibberellin receptor (*MELO3C015362*) maps 400Kbp far from the marker 2:689400-SNV, associated with FLTM. In Arabidopsis, *GID1* homolog mutants are extremely late flowering under long-day conditions and fails to flower under short-day conditions [30, 31]. In addition, a putative *CLEAVAGE STIMULATION FACTOR* (*MELO3C00887*2), whose mutation leads to late flowering in Arabidopsis [32], is located 800 Kbp far from the locus 8:24132601-SNV, also associated with FLTM in this study. A candidate gene was also found for SW, as an homolog of the *MULTICOPY SUPRESSOR OF IRA1* (*MSI1*) gene (*MELO3C006243*), previously associated with seed development [33], resides 490 Kbp far from the SNP locus 6:2464455-SNV. Functional studies targeting candidate genes identified in this study might prove their causal link with phenotypic variation in melon.

## Conclusions

GBS was for the first time applied in *C. melo*. We provide useful information to understand the genetic structure of this species and to protect minor gene pools from genetic erosion. Finally, our results might prompt molecular breeding approaches and be a resource for future studies aiming to link genomic variation with evolutionary and phenotypic traits.

## Methods

### Plant material

A set of 72 accessions of *C. melo* was obtained by local donors, with their prior informed consent, within the framework of the project “Biodiversity of Apulian vegetable species” (Rural Development Programme, European Agricultural Fund for Rural Development, Reg. EC. No. 1698/2005), aimed at the safeguard and characterization of Apulian rural biodiversity. The accessions, corresponding to winter melons (25) and the commercial types ‘*carosello*’ (28) and ‘*barattiere*’ (19) (Additional file 1), are available at the germplasm bank of the Department of Plant, Soil and Food Science of the University of Bari (Italy) and are managed in accordance with the Italian guidelines for the conservation of agricultural biodiversity (http://www.reterurale.it/flex/cm/pages/ServeBLOB.php/L/IT/IDPagina/9580) and the FAO Genebank Standards for Plant Genetic Resources for Food and Agriculture (http://www.fao.org/3/a-i3704e.pdf).

### GBS assay and SNP filtering

Genomic DNA was isolated from young leaf samples using the DNeasy Plant Mini Kit (Qiagen). A reduced representation GBS library was prepared using the restriction enzyme *Ape*KI as described by Elshire et al. [8] and sequenced (single-end reads) using Illumina HiSeq 2500. The TASSEL-GBS pipeline [9] and the melon 3.5.1. genome assembly [13] were used to call SNPs from uniquely aligned reads and generate an hapmap file. Besides default parameters, a depth ranging from 10 to 300 and a minimum quality score of 20 were imposed. Functional and structural annotation of variants were performed using SnpEff 4.2. [34]. Additional filters were applied to select a subset of SNPs for subsequent genetic analyses. In more detail, biallelic SNPs were filtered for minor allele frequency (MAF) higher than 5%, call rate higher than 80% and proportion of heterozygous lower than 50%, using TASSEL v5.2.20 [35]. The filtering procedure was applied on the whole germplasm collection or on each of the three subpopulations identified by structure analysis, depending on the input dataset required for downstream analyses.

### Genetic structure and molecular diversity among individual accessions

The admixture-based clustering model implemented in the software STRUCTURE 2.3.4 [12] was used to estimate the number of hypothetical subpopulations (K) and the probability of individual accessions to fall in each subpopulation. As the STRUCTURE algorithm assumes independent loci, the SNP dataset was pruned prior to analysis based on pairwise linkage disequilibrium (LD) between adjacent markers. This was estimated using the SNP & Variation Suite (SVS) software v8.4.0 (Golden Helix Inc.), setting the threshold for r^2^ equal to 0.5. Each K was run ten times with a burn-in period of 100,000 and 100,000 Markov chain Monte Carlo (MCMC) repeats after burn-in. The value of the ΔK parameter, based on the second order rate of change of the likelihood function (ln Pr (X|K)), was used as criterion to estimate the true K [14]. Genotypes were assigned to one of the subpopulations when the value of the corresponding membership coefficient (q) was higher than 0.6. If not, they were considered admixed.

LD-pruned SNPs were also used to study molecular diversity among individual accessions. Principal component analysis (PCA) was performed using SVS v8.4.0. Furthermore, a Neighbor-Joining cladogram was obtained using the Archaeopteryx visualization tool implemented in TASSEL v5.2.20.

### LD decay

LD decay was evaluated within the three *C. melo* subpopulations identified by structure analysis. Pairwise r^2^ values, estimated using the expectation-maximization (EM) algorithm implemented in SVS v8.4.0, were plotted against the distance (kb) between adjacent SNP loci, and a regression curve was fit to the data.

### *F*_*ST*_ analysis

For each subpopulation pair, fixation index (F_ST_) estimates were obtained using the formula of Weir and Cockerham [36] available in SVS v8.4.0. Confidence intervals around the F_ST_ value were estimated using the percentile-t bootstrapping technique reported by Leviyang and Hamilton [37], implemented by the same software. The average F_ST_ provided a measure of genetic distance between subpopulations. F_ST_ values at individual loci were plotted against the melon 3.5.1. genome assembly to highlight genomic regions putatively subjected to directional selection. SNP loci associated with F_ST_ estimates higher than 0.9 were used to draw a custom Venn diagram.

### Association mapping

Phenotypic traits (FLTM, FLTP, SL, SW, and SWe) were collected on plant material sown at the experimental farm “P. Martucci” of the University of Bari (41°01′22.1″N 16°54′21.0″E), according to a randomized block design with 3 replicates. FLTM and FLTP were recorded as the number of days from the sowing date to the date when 50% of the plants showed the first flower completely open. Cross-pollination among accessions was prevented using net cages in which bumble bees were introduced. SL, SW and SWe were determined on 100 seeds collected on 10 randomly chosen individuals. Association between SNP loci with a minimum MAF of 10% and phenotypes was determined using the weighted mixed linear model (MLM) implemented in TASSEL v5, taking into account kinship and genetic structure (K + Q). Significant associations were inferred using an adjusted p-value (Bonferroni correction).

## Additional files

Additional file 1: Accessions of *Cucumis melo* genotyped in this study. Abbreviation in the origin field refer to different Provinces within the Apulia Region (BA: Bari; BAT: Barletta-Andria-Trani; BR: Brindisi; TA: Taranto; LE: Lecce). (XLSX 78 kb)
Additional file 2: Genomic distribution of the 8,012 high-quality SNPs obtained by GBS analysis of the *C. melo* germplasm collection used in this study. (PPTX 53 kb)
Additional file 3: Delta K distribution from STRUCTURE analysis. K = 3 shows a peak indicating that three sub-populations sufficiently define genetic variation in the *C. melo* germplasm collection considered in this study. (PPTX 48 kb)
Additional file 4: Yellow (a), green (b) and speckled (c) rind phenotype of *C. melo* var. *inodorus* accessions collected in this study. (PPTX 3164 kb)
Additional file 5: Principal component analysis of the I subpopulation. Accessions with yellow, green and speckled rind are represented by yellow, green and blue dots, respectively. (PPTX 41 kb)
Additional file 6: Principal component analysis of the C subpopulation. Accessions originating from the Southern area of Apulia are highlighted with a circle. (PPTX 49 kb)
Additional file 7: List of highly divergent loci (F_ST_ > 0.9) in pairwise comparisons among the subpopulations C, B and I. The name of each locus includes the chromosome, the chromosomal position, and the type of polymorphism (SNV = single nucleotide variation; Ins: single nucleotide insertion; Del: single nucleotide deletion). For SNP loci falling in putative genes sequences, gene ID and functional annotation reported in the Melonomics database (www.melonomics.net) are indicated. Loci which define alleles private to one of the subpopulations are highlighted in red. (XLSX 67 kb)
Additional file 8: Clusters of at least two consecutive markers displaying F_ST_ > 0.9 in pairwise comparisons among the subpopulations B, C and I. (XLSX 9 kb)
Additional file 9: Manhattan plot of the genome-wide association study for flowering time of male flowers (FLTM). Chromosome coordinates are displayed along the X-axis. For each locus, the negative log 10 of the *p*-value for association is displayed on the Y-axis. (BMP 1318 kb)
Additional file 10: Manhattan plot of the genome-wide association study for seed width (SW). Chromosome coordinates are displayed along the X-axis. For each locus, the negative log 10 of the *p*-value for association is displayed on the Y-axis. (BMP 1318 kb)

## Abbreviations

EM: Expectation-maximization
FLMP: Flowering time of perfect flowers
FLTM: Flowering time of male flowers
GBS: Genotyping by sequencing
GWAS: Genome-wide association studies
LD: Linkage disequilibrium
MAF: Minor allele frequency
MCMC: Markov chain Monte Carlo
MLM: Mixed linear model
NGS: Next generation sequencing
PCA: Principal component analysis
SL: Seed length
SNP: Single nucleotide polymorphism
SSR: Simple sequence repeat
SW: Seed width
SWe: 100 seed weight
