# Supplementary figures and images for: Genotyping-by-sequencing of a melon (Cucumis melo L.) germplasm collection from a secondary center of diversity highlights patterns of genetic variation and genomic features of different gene pools
Source: BMC Genomics. 2017 Jan 9;18:59. doi: 10.1186/s12864-016-3429-0 (PMC5223370; doi:10.1186/s12864-016-3429-0)

## Slide 1
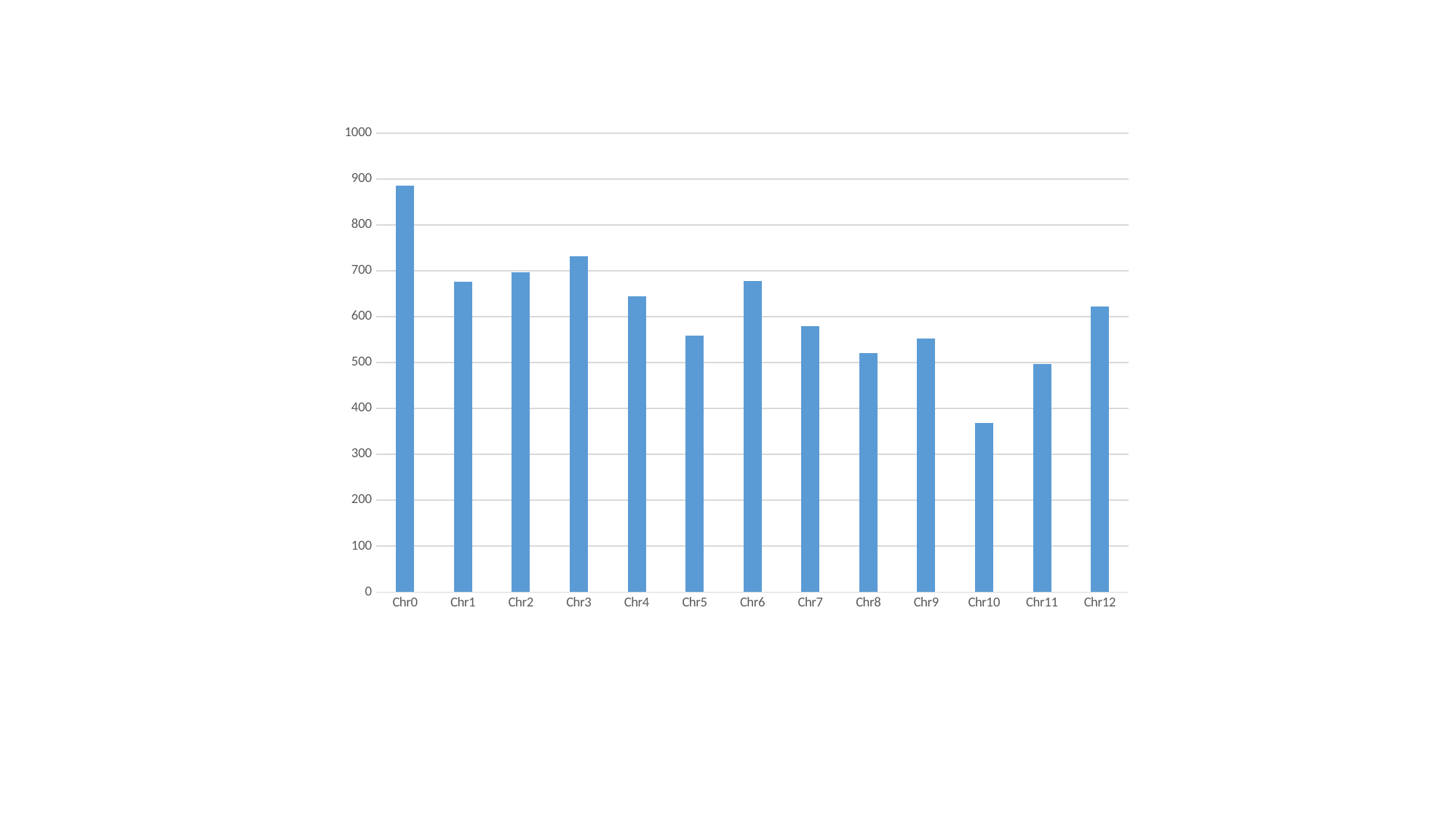

### Chart
| Category | SNP# |
|---|---|
| Chr0 | 886.0 |
| Chr1 | 676.0 |
| Chr2 | 697.0 |
| Chr3 | 732.0 |
| Chr4 | 644.0 |
| Chr5 | 559.0 |
| Chr6 | 677.0 |
| Chr7 | 580.0 |
| Chr8 | 521.0 |
| Chr9 | 552.0 |
| Chr10 | 369.0 |
| Chr11 | 497.0 |
| Chr12 | 622.0 |

Supplement: Additional file 2: — Genomic distribution of the 8,012 high-quality SNPs obtained by GBS analysis of the C. melo germplasm collection used in this study. (PPTX 53 kb) [file 12864_2016_3429_MOESM2_ESM.pptx]

## Slide 1
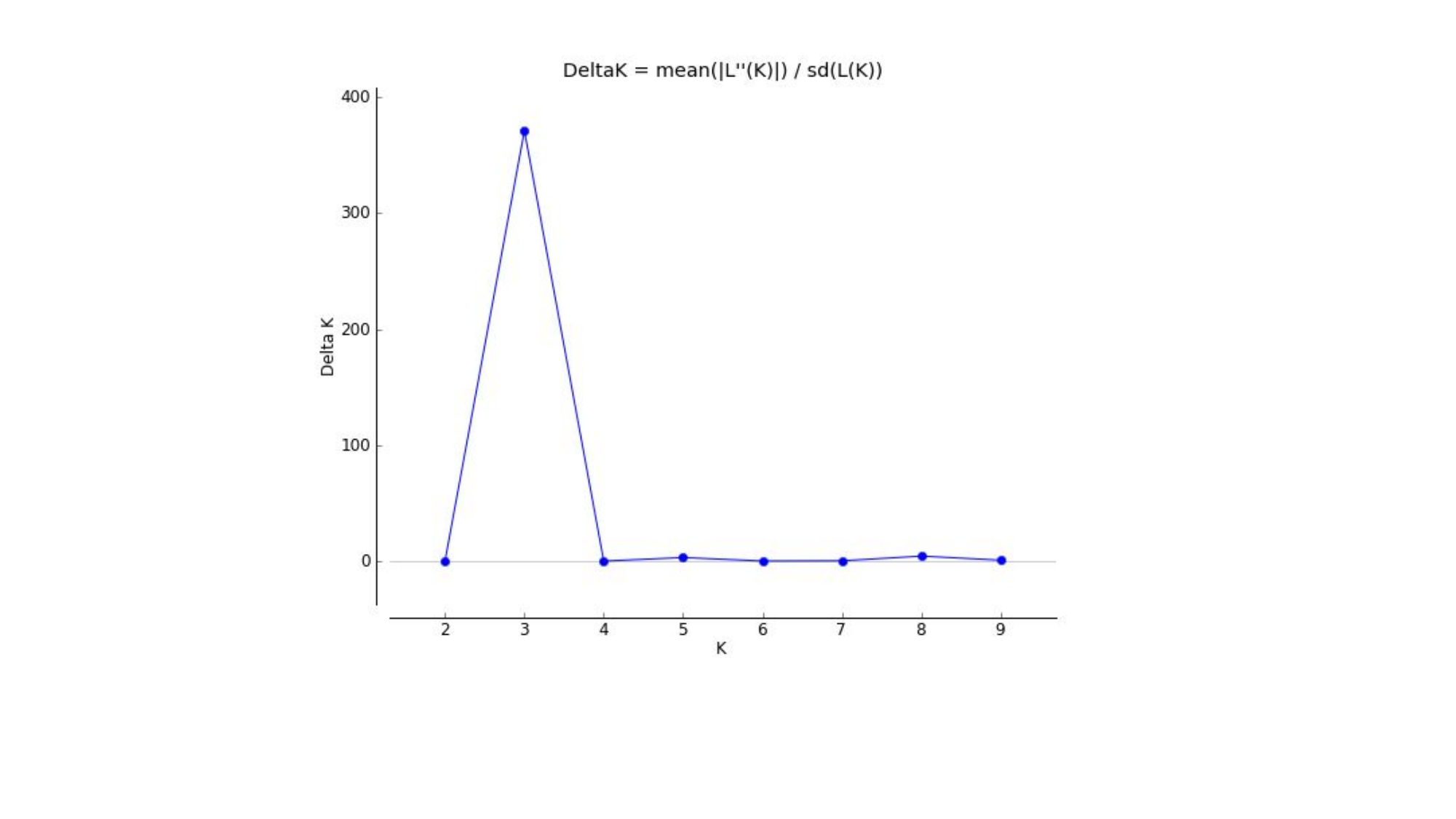

Supplement: Additional file 3: — Delta K distribution from STRUCTURE analysis. K = 3 shows a peak indicating that three sub-populations sufficiently define genetic variation in the C. melo germplasm collection considered in this study. (PPTX 48 kb) [file 12864_2016_3429_MOESM3_ESM.pptx]

## Slide 1
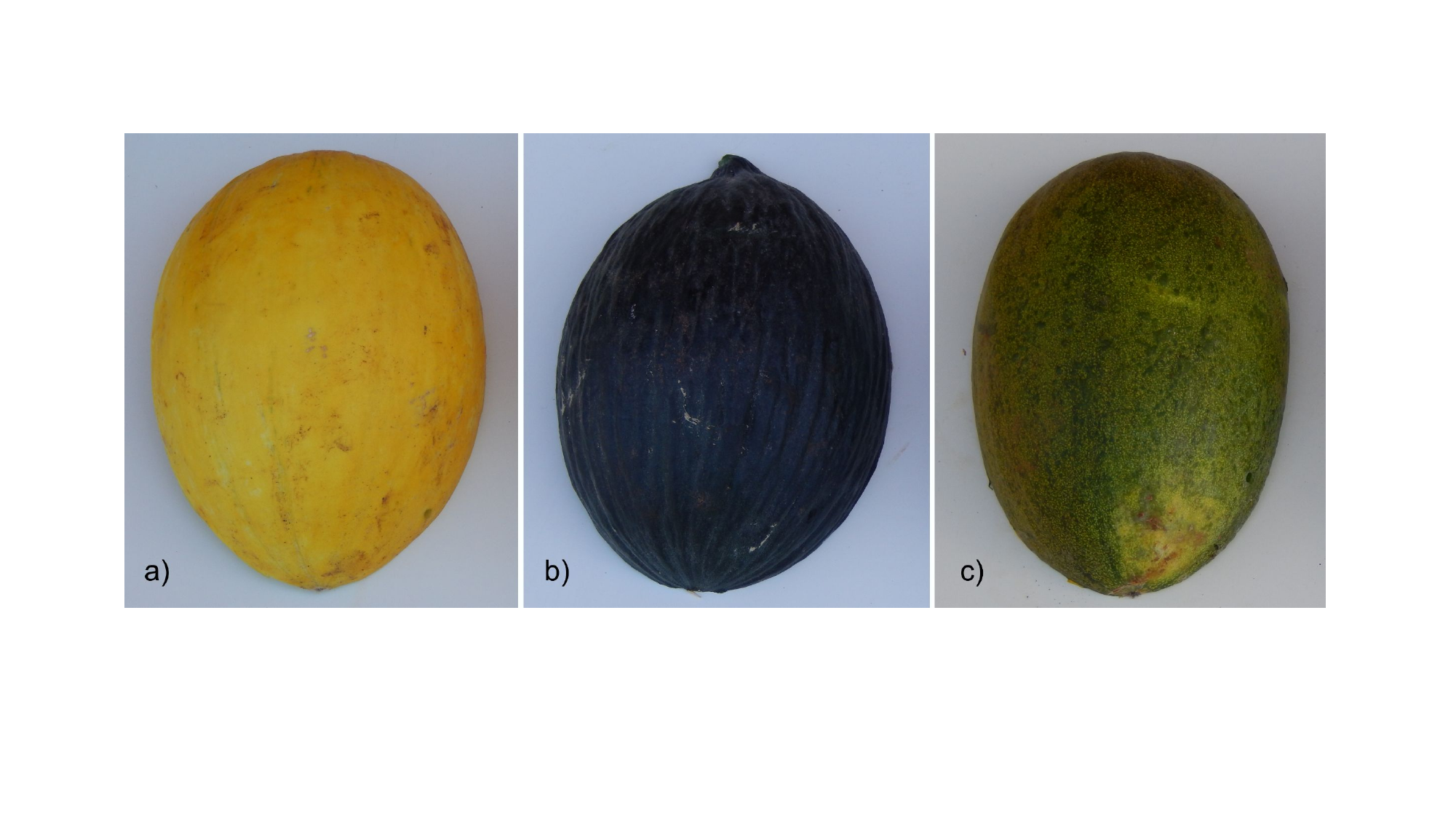

Supplement: Additional file 4: — Yellow (a), green (b) and speckled (c) rind phenotype of C. melo var. inodorus accessions collected in this study. (PPTX 3164 kb) [file 12864_2016_3429_MOESM4_ESM.pptx]

## Slide 1
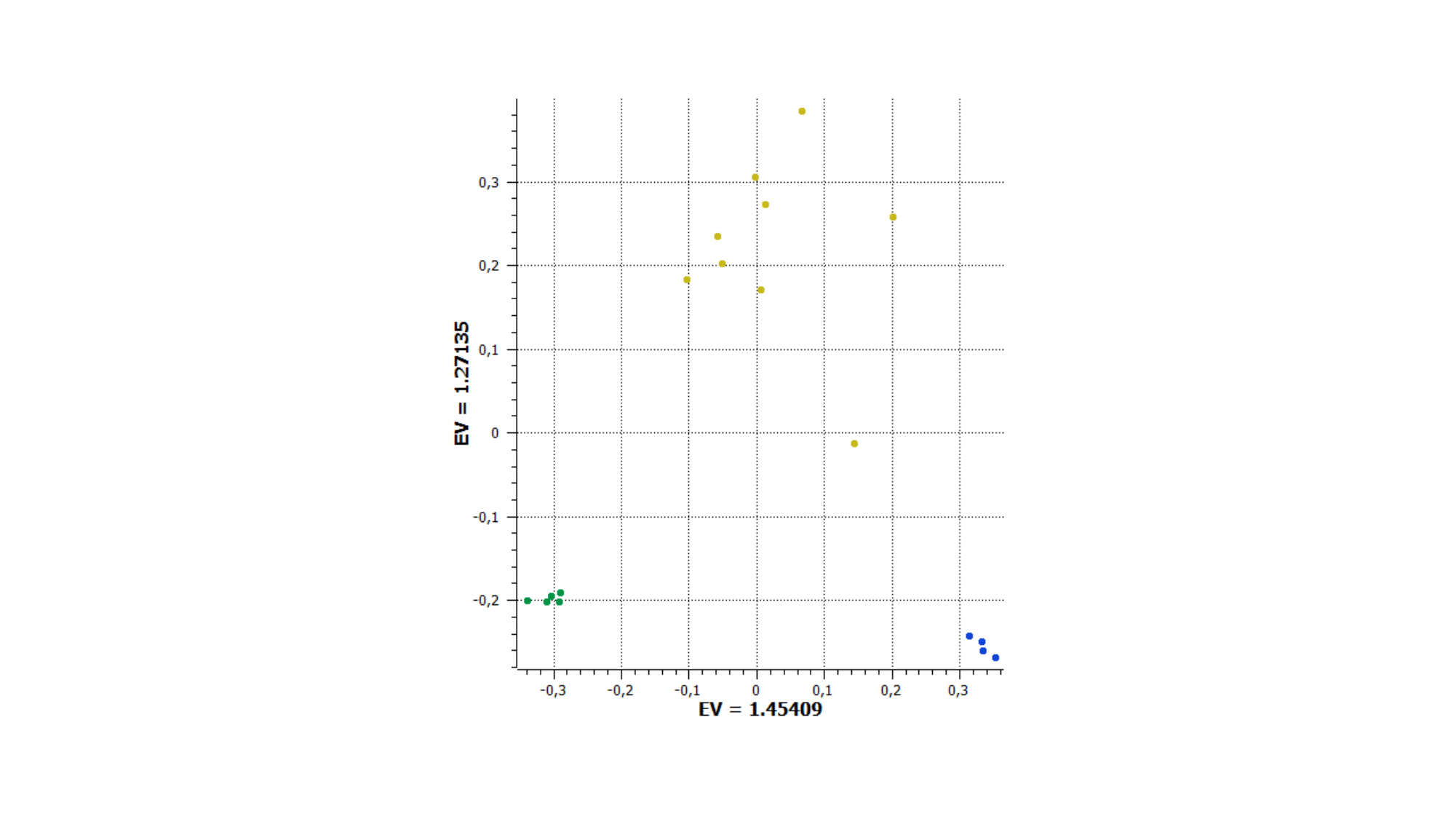

Supplement: Additional file 5: — Principal component analysis of the I subpopulation. Accessions with yellow, green and speckled rind are represented by yellow, green and blue dots, respectively. (PPTX 41 kb) [file 12864_2016_3429_MOESM5_ESM.pptx]

## Slide 1
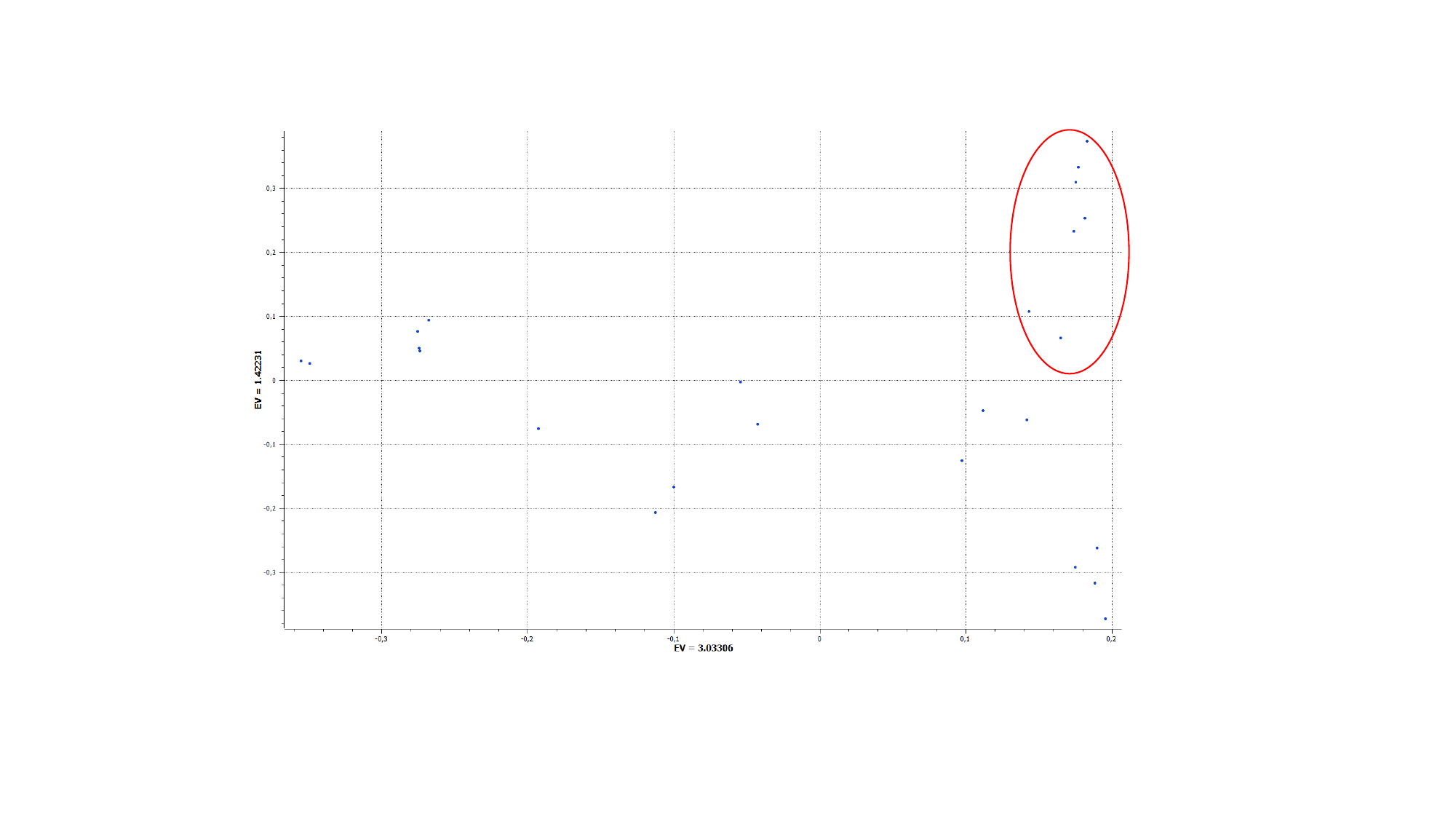

Supplement: Additional file 6: — Principal component analysis of the C subpopulation. Accessions originating from the Southern area of Apulia are highlighted with a circle. (PPTX 49 kb) [file 12864_2016_3429_MOESM6_ESM.pptx]

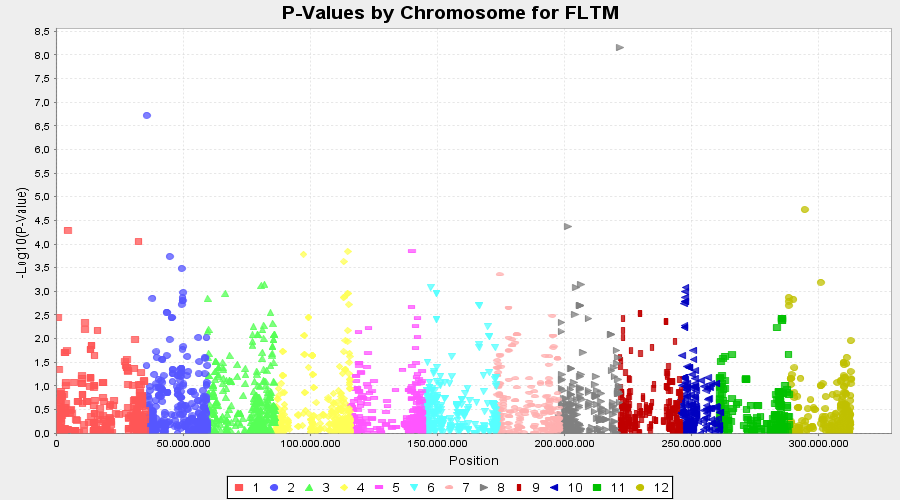

Supplement: Additional file 9: — Manhattan plot of the genome-wide association study for flowering time of male flowers (FLTM). Chromosome coordinates are displayed along the X-axis. For each locus, the negative log 10 of the p-value for association is displayed on the Y-axis. (BMP 1318 kb) [file 12864_2016_3429_MOESM9_ESM.bmp]

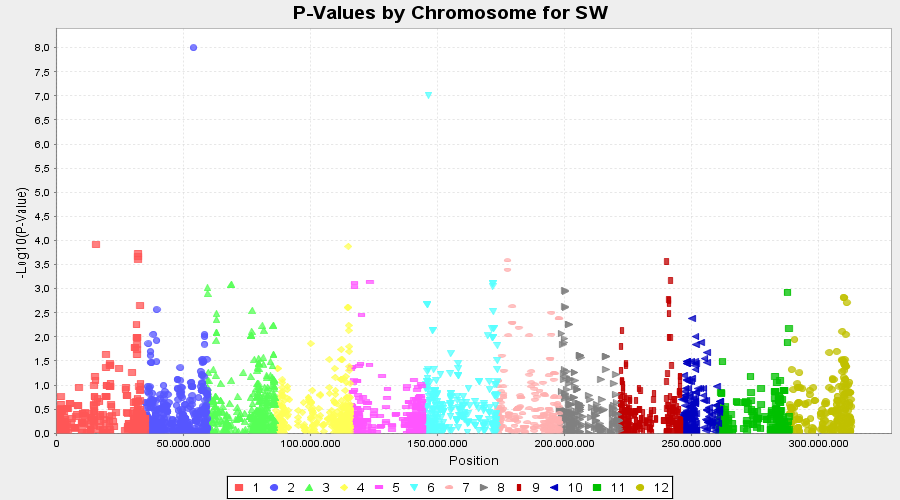

Supplement: Additional file 10: — Manhattan plot of the genome-wide association study for seed width (SW). Chromosome coordinates are displayed along the X-axis. For each locus, the negative log 10 of the p-value for association is displayed on the Y-axis. (BMP 1318 kb) [file 12864_2016_3429_MOESM10_ESM.bmp]
